# Am I sentenced to life-long use of antipsychotics? A qualitative analysis of Q&A data about stopping antipsychotics from the perspective of users and their relatives

**DOI:** 10.1192/j.eurpsy.2025.708

**Published:** 2025-08-26

**Authors:** S. Crutzen, J. van Os, S. Castelein

**Affiliations:** 1 Lentis Research, Lentis Psychiatric Institute, Groningen; 2 UMC Utrecht Brain Centre, University Medical Centre Utrecht, Utrecht; 3 Department of Psychiatry and Neuropsychology, Maastricht University Medical Centre, Maastricht, Netherlands; 4 Institute of Psychiatry, King’s College London, London, United Kingdom; 5 Department of Clinical Psychology and Experimental Psychopathology, University of Groningen, Groningen, Netherlands

## Abstract

**Introduction:**

The majority of antipsychotic users at some point want to stop their antipsychotic medication and many do so without consulting their attending physician. So-called non-adherence to antipsychotics has been estimated to be as high as 60% and it has been identified as the most important predictor for relapse, resulting in a four times higher risk of relapse. When asking antipsychotic users, different reasons for wanting to stop are mentioned. These reasons include severe side effects, reduced functioning, experiencing no benefits and long-term physical health concerns. From the perspective of patients, wanting to stop can be considered an understandable and rational reaction given the burdens that antipsychotic use often imposes. Given the current uncertainties surrounding stopping antipsychotics, and given the patients’ wish to be involved in treatment decisions and a move away from a paternalistic mental health care model, several shared decision initiatives have been formed to involve patients more in the decisions about stopping or reducing the dose of their antipsychotic. In order to support shared decision-making, insight is needed in which questions antipsychotic users have.

**Objectives:**

The current study aims to gain insight in which questions antipsychotic users and their relatives have, and which factors influence stopping or reducing the dose of antipsychotics, by qualitatively analysing questions posted on an online expert Q&A.

**Methods:**

Data were used from a Dutch existing publicly available anonymous expert Q&A. Questions about stopping or reducing the dose of antipsychotics were analysed using an inductive thematic approach. Questions antipsychotic users and their relatives had about this topic and factors that influences the process were identified.

**Results:**

In total 194 out of 3000 screened questions were about stopping or reducing antipsychotic dose. The most common question was whether it was sensible to stop or reduce the dose. Questions focused on how fast to reduce the dose, what their minimum dose should be and where they could find support. Those that were phasing out their antipsychotic asked when withdrawal symptoms or side effects would subside. Motivations to stop were side effects, difficulties in assuming a normal life and social roles and experiencing no benefits. Barriers were lack of support and return of symptoms. Facilitators were support from others and experiencing a relief from side effects and/or symptoms. Finally, questions were asked about activities that might support discontinuation.

**Conclusions:**

Antipsychotic users continue to be left with many questions about stopping or reducing the dose of antipsychotics. These questions reveal attitudes, preferences and concerns regarding antipsychotic treatment that are important to address when discussing antipsychotic treatment.

**Disclosure of Interest:**

None Declared

